# Novel use of an exchange catheter to facilitate intubation with an Aintree catheter in a tall patient with a predicted difficult airway: a case report

**DOI:** 10.1186/1752-1947-6-108

**Published:** 2012-04-13

**Authors:** Shaun E Gruenbaum, Benjamin F Gruenbaum, Sergey Tsaregorodtsev, Michael Dubilet, Israel Melamed, Alexander Zlotnik

**Affiliations:** 1 Department of Anesthesiology, Yale University School of Medicine, New Haven, CT, USA; 2 Department of Anesthesiology and Critical Care, Soroka Medical Center and Ben Gurion University of the Negev, Beer Sheva, Israel; 3 Department of Neurosurgery, Soroka Medical Center and Ben Gurion University of the Negev, Beer Sheva, Israel

## Abstract

**Introduction:**

The Aintree intubating catheter (Cook^® ^Medical Inc., Bloomington, IN, USA) has been shown to successfully facilitate difficult intubations when other methods have failed. The Aintree intubating catheter (Cook^® ^Medical Inc., Bloomington, IN, USA) has a fixed length of 56 cm, and it has been suggested in the literature that it may be too short for safe use in patients who are tall.

**Case presentation:**

We present the case of a 32-year-old, 180 cm tall Caucasian woman with a predicted difficult airway who presented to our facility for an emergency cesarean section. After several failed intubation attempts via direct laryngoscopy, an airway was established with a laryngeal mask airway. After delivery of a healthy baby, our patient's condition necessitated tracheal intubation. A fiber-optic bronchoscope loaded with an Aintree intubating catheter (Cook^® ^Medical Inc., Bloomington, IN, USA) was passed through the laryngeal mask airway into the trachea until just above the carina, but was too short to safely allow for the passage of an endotracheal tube.

**Conclusions:**

We present a novel technique in which the Aintree intubating catheter (Cook^® ^Medical Inc., Bloomington, IN, USA) was replaced with a longer (100 cm) exchange catheter, over which an endotracheal tube was passed successfully into the trachea.

## Introduction

The Aintree intubating catheter (AIC) (Cook^® ^Medical Inc., Bloomington, IN, USA) has been demonstrated to successfully enable fiber-optic-guided intubation through a laryngeal mask airway (LMA) when conventional attempts at tracheal intubation have failed. The AIC has a fixed length of 56 cm and 4.8 mm diameter lumen but is intended for patients of all sizes. Here, we describe a case of a difficult intubation in which an AIC was inserted through an LMA, however the AIC was too short for an endotracheal tube (ETT) to be passed while simultaneously maintaining the position of the AIC. A novel approach is described, in which the AIC was carefully replaced by a longer exchange catheter, over which an ETT was successfully passed into the trachea.

## Case presentation

A 32-year-old Caucasian woman presented to our facility at 39 weeks gestation for an emergency cesarean section due to prolapse of the umbilical cord and fetal distress. The course of her pregnancy had been uneventful and our patient was admitted after the onset of regular contractions. When the cervix was dilated at 5 cm, the amniotic membranes ruptured and the fetus quickly developed signs of fetal distress, including fetal bradycardia down to 100 beats/minute and variant decelerations. A vaginal examination revealed prolapse of the umbilical cord.

Our patient was immediately brought to the operating room for an urgent cesarean section. A pre-operative anesthetic examination did not reveal any coexisting medical conditions, though she had a predictive difficult airway: Mallampati score III, thyromental distance measured 4 cm, no prominent incisors, limited neck extension and limited mouth opening (3 cm). Her blood pressure measured 110/70, heart rate 80 beats/minute, pulse oximetry (saturated O_2 _(SaO_2_)) was 99% on room air. Our patient was 180 cm tall and weighed 100 kg. She was denied food and water for nine hours for food and three hours for clear liquids. She refused an epidural catheter.

Considering the urgency of the surgery due to severe fetal distress it was decided to proceed immediately with general anesthesia via rapid sequence induction. Considering the risk of difficult intubation, the difficult intubation cart was brought and an attending anesthesiologist experienced in the difficult airway management of patients in childbirth was called in. An attempt to perform a conscious fiber-optic intubation was considered but not performed because of the urgency of the situation and 'pressure' from the obstetricians. Our patient was attached to standard monitors, including pulse oximetry, non-invasive blood pressure, electrocardiogram (ECG), end-tidal carbon dioxide (ETCO_2_) analyzer, and nerve stimulator.

Our patient received aspiration prophylaxis with metoclopramide 10 mg intravenously prior to induction. After a short pre-oxygenation step (four deep breaths), anesthesia was induced with propofol 2 mg/kg and succinylcholine 1 mg/kg. Cricoid pressure was applied immediately after the injection of propofol. Direct laryngoscopy (DL) was then attempted using a Macintosh (number 3 blade). The first attempt at intubation was performed by an experienced resident, and was unsuccessful because of an inability to visualize the vocal cords (grade III view) and an inability to pass the ETT blindly. Mask ventilation with 100% oxygen was performed easily, and SaO_2 _remained 99%. The second and third attempts at DL were made unsuccessfully by the attending anesthesiologist. All attempts to improve visualization, including repositioning the head and the use of a laryngoscope with Miller's blade, were unsuccessful. Mask ventilation was applied between the attempts at intubation.

After the third attempt, an LMA size 4 was successfully placed and positive pressure ventilation was applied with following parameters: tidal volume (TV) 500 mL, respiratory rate (RR) 12 breaths/minute, positive-end expiratory pressure (PEEP) 0, inspiratory/expiratory (I/E) ratio 1:2. Peak inspiratory pressure did not exceed 25 cm H_2_O and no leak was detected. Cricoid pressure was maintained during the entire procedure to prevent possible aspiration. Anesthesia was maintained with isoflurane 1.2% in pure oxygen. 100% oxygen was maintained to optimize the conditions if the airway was lost. The surgical incision was made immediately after the insertion of the LMA and the fetus was delivered 10 minutes later. Apgar scores were 7 and 10 at the first and 10th minute, respectively.

Our patient remained hemodynamically stable during the surgery, though her SaO_2 _decreased gradually, reaching 90% by the end of the surgery. Her trachea was suctioned, revealing the accumulation of a significant amount of blood in both the pharynx and trachea. Her pulse oximetry (SpO_2_) did not improve after suctioning, and her peak inspiratory pressure increased by 30 cm H_2_O, suggesting aspiration of blood and possibly of gastric contents. The decision was made to intubate our patient in order to facilitate suctioning the tracheobronchial tree and providing ventilatory support for respiratory failure.

**Figure 1:**
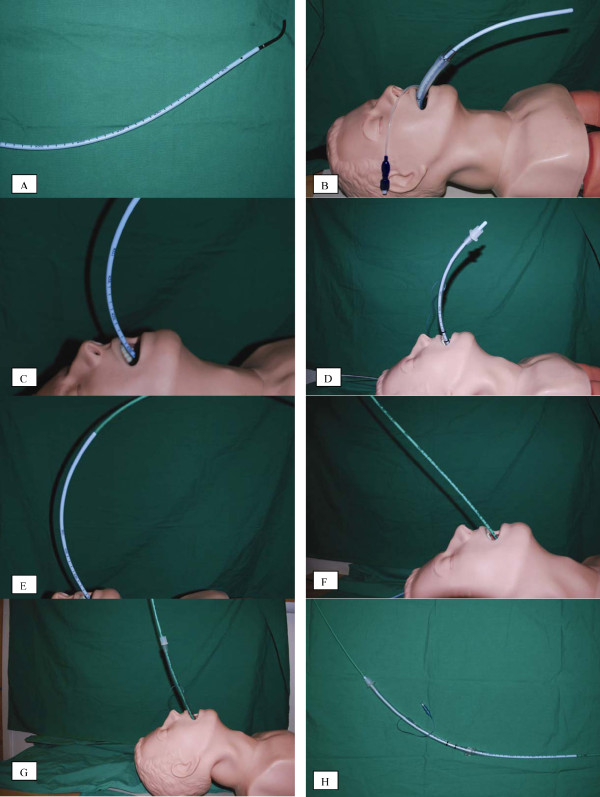
**Steps in the technique, in sequence**. **(A) **Aintree intubating catheter (AIC) (Cook^® ^Medical Inc., Bloomington, IN, USA) was dressed over a flexible fiber-optic bronchoscope (FOB), leaving the distal 4 cm free to allow for flexion and extension of the FOB. **(B) **The FOB with the AIC were passed between the grids of the laryngeal mask airway (LMA) and through the larynx and into the trachea. The FOB was then removed with the AIC left in place. **(C) **The LMA was removed while the position of the AIC was maintained. **(D) **We attempted to 'railroad' the endotracheal tube (ETT) over the AIC, but the length of the portion of the AIC that remained outside our patient after its insertion was shorter than the length of the ETT. We were therefore unable to slide the ETT down into the trachea while maintaining the position of the AIC. **(E) **The exchange catheter was inserted through the inner port of the AIC and advanced until resistance was felt. Note the relative lengths of the two catheters. **(F) **The AIC was removed while the position of the exchange catheter was maintained in the trachea. **(G) **An ETT was advanced over the exchange catheter and into the trachea. The exchange catheter was then removed, and proper positioning of the ETT was confirmed by end-tidal carbon dioxide (ETCO_2_) and via fiber-optic visualization. **(H) **Relative lengths of the ETT, AIC, and exchange catheter when viewed together. The diameter of the exchange catheter is such that it can easily pass through the inner port of the AIC.

Considering that intubation under DL was unsuccessful and fiber-optic intubation with a flexible fiber-optic bronchoscope (FOB) would be extremely difficult due to the presence of upper airway bleeding and swelling from traumatic attempts at intubation, the decision was made to intubate using an AIC dressed over a FOB, via LMA *in situ*. The procedure was performed by the attending anesthesiologist with experience in difficult intubations with patients who are pregnant. An AIC (19 F/56 cm; Cook Inc., Bloomington, IN, USA) was placed on a FOB (Olympus model LF-2 with an external diameter of 4.0 mm). Continuous fresh gas flow of oxygen at 4 L/minute was maintained via the suction port of the FOB in order to maintain better oxygenation and improve visualization by blowing out the blood bubbles. After suctioning the LMA, the FOB was easily passed between the grids of the LMA, the epiglottis and vocal cords were visualized, and the FOB was passed between the vocal cords into the trachea and introduced almost until the tracheal bifurcation. The AIC was then slid down over the FOB, and the FOB was removed (Figure 1B, C).

We attempted to 'railroad' a cuffed ETT with an internal diameter of 6.5 mm down to the trachea using the AIC as a guide. Because our patient was tall, a significant length of the AIC had to be inserted into our patient in order for the distal tip of the AIC to be positioned just before the carina. This left only a short length of the proximal portion of the AIC available for the ETT to be 'railroaded' over (the portion of the AIC that was outside our patient). However, this free portion of the AIC was shorter than the ETT. Therefore, the ETT could not be safely slid over the AIC while simultaneously holding the proximal end of the AIC to maintain its position and prevent its slipping from within the trachea. (Figure 1D). Additionally, the catheter was very slippery because of our patient's secretions and blood. Our patient's SaO_2 _dropped to 80%. Attempts to introduce the ETT were postponed to allow for mask ventilation, and the ETT was removed while the position of the AIC was maintained.

After two minutes of mask ventilation, when the SaO_2 _reached 90%, another attempt at introducing the ETT was made. With this attempt, however, a novel approach was used to create a longer guide as illustrated in Figure 1. After positioning of AIC via LMA with FOB (Figure 1A-D). The AIC was replaced with an exchange catheter for a double-lumen ETT (14 F/100 cm, Cook Inc.). The exchange catheter was lubricated and inserted into the trachea via the inner port of AIC until mild resistance of the tracheobronchial tree was felt (60 cm deep) (Figure 1E). The AIC was slowly removed over the exchange catheter, with careful attention made to keep the exchange catheter in place (Figure 1F). Fortunately, this was easily accomplished because the exchange catheter was very long.

After the Aintree catheter was removed, a cuffed ETT size 6.5 was easily introduced into the trachea over the exchange catheter until 21 cm deep, and the exchange catheter was removed (Figure 1G). The cuff of the ETT was inflated and mechanical ventilation was resumed with the following parameters: TV 500 mL, RR 12 breaths/minute, PEEP 7.5 cm H_2_O, I/E ratio 1:2, fraction of inspired O_2 _(FiO_2_) 1.0. Our patient's peak inspiratory pressure measured 30 cm H_2_O (including PEEP). The position of the ETT was verified by both the appearance of ETCO_2 _waveform on the monitor and via bronchoscopy (the tip of the ETT was observed 3 to 4 cm above the carina). The bronchoscopy revealed traces of blood in the trachea, which was suctioned via the suction port of the bronchoscope. No food particles were detected in the trachea. Her SaO_2 _level gradually reached 99%. Analysis of arterial blood revealed moderate hypercapnia (partial pressure CO_2 _(pCO_2_) of 50 mmHg) and relative hypoxemia (partial pressure of O_2 _in blood (PaO_2_) of 110 mmHg).

Considering our patient's risk of developing aspiration pneumonitis and upper airway edema, sedation with a propofol drip at a dose of 3 mg/kg/hour was initiated and our patient was transferred to the intensive care unit. Fortunately, our patient remained hemodynamically stable and was well oxygenated and ventilated. Our patient did not receive any antibiotics or steroids. She was subsequently extubated 24 hours later, after performing a leak test to rule out pharyngeal or vocal cord edema. No additional complications were noted and our patient was discharged home without any sequelae 48 hours after she was extubated.

## Discussion

Since the introduction of the American Society of Anesthesiologist's (ASA) difficult airway algorithm in 1991 [1,2], there has been much focus on airway management when conventional methods of establishing an airway have failed. Since that time, the LMA has been established as a life-saving tool for establishing an airway in patients whose tracheas cannot be intubated [3]. In recent years, the role of the LMA has been more clearly defined, and its use has been extended to facilitate tracheal intubations.

For situations in which tracheal intubation is necessary, and conventional methods have failed, the AIC can be an important tool for intubation. The AIC, first described by Atherton *et al*. in 1996 [4], is a ventilation-exchange bougie that can be mounted on a FOB to facilitate tracheal intubation with an ETT through an established airway. The AIC was designed for use with a classic (c) LMA, but has also been successfully used and advocated with a ProSeal LMA^® ^[5,6]. The FOB can be passed through the LMA and into the trachea with continued ventilation through the dedicated airway. The LMA can then be safely removed with the AIC remaining in the trachea, and an ETT can then be passed over the AIC and into the trachea. The device is commercially available in one size for all patients: 19 French, 56 cm long with an internal and external diameter of 4.7 mm and 7.0 mm, respectively. The AIC fits well over a 4 mm FOB and allows for 4 cm free at the distal end to enable flexing and extending the FOB (see Figure 1A).

For situations that require a secure airway, tracheal intubation facilitated by an AIC can be accomplished quickly and confidently by anesthetists with little experience in its use [7]. The AIC has been used for tracheal intubation in the context of patients who are awake sedated and in airway emergencies [5,8]. The device has been used successfully in the context of high-risk patients with an anterior mediastinal mass [9], patients with cervical spine pathology with limited neck mobility [10], and patients who are extremely obese [11]. Many centers that use the AIC routinely report a success rate that approaches 100% with a low risk of complications [12-14]. At this time, tracheal intubation facilitated by an AIC is considered a rescue method that could be considered when other methods have failed [14,15].

Although the AIC has been shown to be an invaluable tool in managing a difficult intubation, it has been subject to some criticism. At 56 cm, the fixed length of the AIC has been criticized by some as being too short to allow for reliable exchange of an LMA with ETT [13]. However, it is important to note that some have argued that that the length of the AIC is adequate [12]. The FOB has a fixed length, and when used with the AIC, the distal 4 cm are left free to allow for flexion and extension of the FOB. For this reason, the length of the AIC cannot be increased (Figure 1A). In the present report, we describe the case of a tall patient with a difficult airway in whom the AIC was too short in length to be used safely. We present a novel approach in which an AIC was successfully replaced with a longer exchange catheter for a double-lumen ETT. The ETT was placed over the exchange catheter and successfully passed into the trachea (the location confirmed by the presence of ETCO_2 _and by fiber-optic visualization).

Thus, for a tall patient, to prevent the inadvertent removal or displacement of the AIC out of the trachea it is safe to leave 23 to 25 cm in length from the point just above the carina to the incisors. Therefore, only approximately 31 cm proximal to the incisors remains over which the ETT must be placed. The length of an ETT with an internal diameter of 7 mm and 8 mm is 31 cm and 32 cm in length, respectively. Therefore, little if any length is free to hold the AIC in place while simultaneously sliding the ETT over it. Moreover, the tip of AIC is very slippery due to secretions and applied lubricant, and is therefore difficult to hold in place. The exchange catheter, however, is 100 cm in length and provides plenty of distance to successfully introduce the ETT into the trachea without any significant risk of displacement or inadvertent removal of the guide (Figure 1H).

## Conclusions

In summary, the use of the AIC may be limited in tall patients because its length may be too short to facilitate safe passage of the ETT while simultaneously maintaining the position of the AIC. We described a novel technique of replacing an AIC with an exchange catheter over which an ETT was successfully passed into the trachea. This approach can be utilized when necessary to facilitate an AIC in tall patients with a difficult airway in whom conventional methods of tracheal intubation have failed.

## Consent

Written informed consent was obtained from the patient for publication of this case report and any accompanying images. A copy of the written consent is available for review by the Editor-in-Chief of this journal.

## Competing interests

The authors declare that they have no competing interests.

## Authors' contributions

ST, MD, IM, and AZ were jointly involved in the care of our patient. SG, BG and AZ were responsible for the conception and design, analysis and interpretation of the case, as well as were major contributors in writing the manuscript. All authors read, provided critical revisions and approved the final manuscript.
